# Intention to Seek Mental Health Services During the 2022 Shanghai COVID-19 City-Wide Lockdown: Web-Based Cross-Sectional Study

**DOI:** 10.2196/51470

**Published:** 2024-12-02

**Authors:** Lingzi Luo, Gen Li, Weiming Tang, Dan Wu, Brian Hall

**Affiliations:** 1 New York University School of Global Public Health New York, NY United States; 2 New York University Shanghai Center for Global Health Equity Shanghai China; 3 University of North Carolina at Chapel Hill Project-China Guangzhou China; 4 Nanjing Medical University Nanjing China

**Keywords:** COVID-19, mental health services, intention, mobile, digital, lockdowns, depression, anxiety, help-seeking, regression, applications, mHealth, WeChat, pandemic, social isolation, mental health, intent, outbreak, SARS-CoV-2, survey, usage, service

## Abstract

**Background:**

The implementation of COVID-19 lockdown measures had immediate and delayed psychological effects. From March 27, 2022, to June 1, 2022, the Shanghai government enforced a city-wide lockdown that affected 25 million residents. During this period, mental health services were predominantly provided through digital platforms. However, limited knowledge exists regarding the general population’s intention to use mental health services during this time.

**Objective:**

This study aimed to assess the intention of Shanghai residents to use mental health services during the 2022 Shanghai lockdown and identify factors associated with the intention to use mobile mental health services.

**Methods:**

An online survey was distributed from April 29 to June 1, 2022, using a purposive sampling approach across 16 districts in Shanghai. Eligible participants were adults over 18 years of age who were physically present in Shanghai during the lockdown. Multivariable logistic regression was used to estimate the associations between demographic factors, lockdown-related stressors and experiences, physical and mental health status, and study outcomes–mobile mental health service use intention (mobile applications and WeChat Mini Programs [Tencent Holdings Limited]).

**Results:**

The analytical sample comprised 3230 respondents, among whom 29.7% (weighted percentage; n=1030) screened positive for depression or anxiety based on the 9-item Patient Health Questionnaire or the 7-item Generalized Anxiety Disorder Scale. Less than one-fourth of the respondents (24.4%, n=914) expressed an intention to use any form of mental health services, with mobile mental health service being the most considered option (19.3%, n=728). Only 10.9% (n=440) used digital mental health services during the lockdown. Factors associated with increased odds of mobile mental health service use intention included being female, being employed, being a permanent resident, experiencing COVID-19–related stressors (such as loss of income, food insecurity, and potentially traumatic experiences), and having social and financial support. Individuals with moderate or severe anxiety, as well as those with comorbid anxiety and depression, demonstrated a higher intention to use mobile mental health services. However, individuals with depression alone did not exhibit a significantly higher intention compared with those without common mental disorders.

**Conclusions:**

Despite a high prevalence of common mental disorders among Shanghai residents, less than one-fourth of the study respondents expressed an intention to use any form of mental health services during the lockdown. Mobile apps or WeChat Mini Programs were the most considered mental health service formats. The study provided insights for developing more person-centered mobile mental health services to meet the diverse needs of different populations.

## Introduction

The COVID-19 outbreak is one of the most severe public health threats in the past few decades. Large-scale preventative measures such as lockdowns or personal quarantines have been implemented worldwide to contain the spread of the disease. During lockdowns, individuals may experience stressors such as loss of income [1,2], food insecurity [3,4], grieving for lost loved ones [5], and increased risk of violence given the stressors and prolonged periods spent at home [6-8]. Lockdowns and quarantines are associated with negative psychological outcomes, such as anxiety [9,10], depression [10,11], stress [10], and poor sleep health [12]. Epidemiological studies reported that the prevalence of common mental disorders reached 20%-30% among individuals who experienced long-term lockdown or quarantine, highlighting the increased public mental health burden during these stressful COVID-19 control measures [13,14].

In low- and middle-income countries (LMICs), the lack of mental health care infrastructure and shortage of mental health professionals limits the availability of professional options to cope with COVID-19–related stressors. With large-scale face-to-face interventions infeasible, digital mental health services have been considered desirable or necessary alternatives, but public acceptance of digital mental health services is not guaranteed. A prepandemic scoping review of 76 studies found that the general public perceived digital mental health services as less helpful and had lower intentions to use them compared to traditional face-to-face interventions [15]. As health care delivery increasingly shifts towards digital platforms during the pandemic, public acceptance of digital mental health services is likely to have increased. Studies conducted in high-income countries such as the United States and Canada showed increased use of digital mental health tools among the general public, with as high as 70.2% of participants reported routinely using digital mental health support [16], but there is limited research in understanding the general public’s intention and actual use of digital mental health services in LMICs.

On March 27, 2022, the Shanghai government announced a 2-phase 4-day lockdown, which was quickly expanded city-wide and extended to June 1, 2022. This provides a unique opportunity to understand residents’ intentions and use of digital health services during a lockdown for several reasons. China has among the most strictly implemented lockdown measures worldwide, with established policy and service infrastructure to support remote mental health services. The National Health Commission of China published several guidelines for emergency mental health crisis interventions, including provisions for digital mental health services such as mental health hotlines [17]. Mental health associations and academic societies provided free crisis hotlines and online mental health services [18,19], and digital mental health interventions have been made available for residents confined at home [9,20-22]. In the meantime, citizen-government collaboration became prevalent [23]. Self-organized groups emerged to provide emotional, financial, and food support to the residents because of the magnitude of a city-wide lockdown, making it possible to understand the association between COVID-19–related stressors, support, and digital mental health service intention. In addition, the market dominance of social media apps in China, such as WeChat, with 1.2 billion active accounts [24,25], provides innovation potential, such as building widgets embedded in social media apps (WeChat Mini Programs) without the need for additional application downloads.

Previous research examined the association between mental health service use intention and individual demographic characteristics and socioeconomic status [26], mental health symptom severity [27], past help-seeking behaviors [28], perceived social support [29,30], and mental health stigma [31]. More research is needed to understand how COVID-19 related stressors and experiences may impact service use intention and use during lockdowns in addition to these established factors. As digital mental health service use may be constrained by service availability or other additional barriers, the primary interest of this study is to understand mobile mental health service intention to inform future research and product development to fill in the intention and use gap. This study has two aims: (1) to provide an overall description of mental health service intention by service type or provider and the proportion of respondents that used digital mental health services; (2) to examine factors associated with mobile mental health services use intention during the 2022 lockdown.

## Methods

### Participant Recruitment and Eligibility

This study recruited participants between April 29, 2022, and June 1, 2022, from the middle to the end of the lockdown period. An online questionnaire in Chinese was distributed using Wenjuanxing, the most widely used online data collection platform in China [32]. The study used a purposive sampling approach, aiming to recruit 200 residents from each of Shanghai's 16 districts. Network IP addresses were used to identify potential participants residing in Shanghai and their corresponding geographic districts. Each district was capped at 220 respondents, with a 10% oversampling to account for potential invalid responses. The eligibility criteria included adults over 18 years old who lived in Shanghai at the time of the survey. A detailed description of the sampling method is published elsewhere [13].

### Measures and Instruments

#### Sociodemographic Information

Participants reported their age, gender, ethnicity, highest level of education, marital status, family income level before the lockdown, employment status at the time of the survey completion, and migrant status. Migration status included 4 categories: Shanghai native (born in Shanghai), permanent migrant with Shanghai hukou (residency registration), permanent migrant without Shanghai hukou, and temporary migrant (those planning to move to other cities or return to their hometown). Participant hukou status was then categorized into a binary variable, with native Shanghai locals and permanent migrants with hukou labeled as “0” and temporary and permanent migrants without hukou labeled as “1.”

#### Stressors Experienced During Lockdown

Participants were asked to identify the stressors they faced during the lockdown, including loss of income, food insecurity, and potentially traumatic experiences. Loss of income was assessed at 5 levels (did not lose, less than 25%, 26%-50%, 51%-75%, and more than 75%). Food insecurity was assessed with 5 items adapted from the Household Food Insecurity Access Scale (HFIAS) [33,34]. Based on the HFIAS standard scoring, participants were categorized into no, mild, moderate, and severe food insecurity levels. Potentially traumatic events in lockdown were assessed in two domains: (1) witnessing (with one’s own eyes) serious illness or death of a loved one during the lockdown (yes/no) and (2) encountering domestic violence. Domestic violence was assessed using a translated version of the Extended-Hurt/Insult/Threaten/Scream (E-HITS) tool [35]. The E-HITS tool is a 5-item validated instrument to assess physical abuse, insult, threaten with harm, scream or curse, and sexual abuse on a 5-point scale (never, rarely, sometimes, often, or very often) [35,36], and has been applied in the Chinese population [37]. Potentially traumatic event exposure during lockdown was a dichotomized yes/no variable; participants who reported witnessing serious illness or death of a loved one in lockdown or reported at least “sometimes” among any domestic violence items were categorized as having potentially traumatic experiences during lockdown. Participants were also asked whether they had lost a loved one in the past 12 months. In the final analysis, potentially traumatic experiences (PTEs) had 3 categories: no or mild PTE exposure, loss of a loved one in the past 12 months only, and PTE exposure during lockdown.

#### External Support During Lockdown

Participants reported the frequency of emotional support, financial support, and food support received from the government, employer, neighbors, volunteers, family and friends, roommates, or other sources. Each type of support was dichotomized as “yes” or “no” based on whether participants reported ever receiving such support during the lockdown.

#### Physical and Mental Health Status

Self-rated health was assessed through a single-item question [38,39]. Participants’ physical health status was categorized as excellent or very good or good versus fair or poor. Mental health status included depression and anxiety assessed using the Chinese version of the 9-item Patient Health Questionnaire (PHQ-9) [40] and the Chinese version of the 7-item Generalized Anxiety Disorder Scale (GAD-7), respectively [41,42]. These were validated instruments assessing symptom severity over the last 2 weeks on a 4-point scale. Depression and anxiety status was dichotomized into “yes” or “no” with a cut-off score of 10 or above (moderate or severe symptoms) as “yes” for both the PHQ-9 and the GAD-7 (Cronbach α=0.89 for PHQ-9; Cronbach α=0.92 for GAD-7) [40,42].

#### Mental Health Service Use Intention

Mental health service use intention was assessed with a list of varied mental health services or providers being considered during the lockdown period. The list of mental health services was adapted from the General Help-seeking Questionnaire [43]. It included mental health professionals (such as psychiatrists, psychologists, social workers, or mental health counselors); other staff or professionals in mental health service organizations; private mental health clinics; mental health helpline; mobile mental health (mobile app or WeChat Mini Programs), or other services. The responses were dichotomized as “yes” or “no” for each item and, for the composite outcome, any mental health service use intention.

#### Digital Mental Health Service Use During Lockdown

Digital mental health service use during lockdown was assessed with 1 survey item asking if respondents had used any remote mental health services such as hotlines, mobile apps, WeChat mini programs, or online classes.

### Statistical Analysis

The study constructed inverse probability weights based on the sample data and the 2020 Shanghai Census Data [44,45]. All analyses incorporated the survey weights to increase data representativeness. Descriptive statistics were reported using raw frequencies and weighted percentages. Bivariable associations between study covariates and the primary outcome, mental health service use intention, were assessed with 2-tailed chi-square tests or Fisher exact test for any group with less than 5 participants in subgroups. Multivariable logistic regression analysis was performed to estimate the adjusted association between participants’ demographics and socioeconomic status, stressors experienced during lockdown, external support, physical and mental health, and their intention to use mental health services. These factors were chosen either as primary interests or based on previous research and our data indicating they are either potential confounders or effect modifiers.

Data were analyzed using Stata/SE 17.0 (StataCorp), with a threshold for statistical significance at *P*<.05 [46]. The logistic regression model’s goodness of fit was assessed using the “estat” and “roctab” command, the model was also diagnosed with the “linktest” command [46]. The study analysis followed the STROBE (Strengthening the Reporting of Observational Studies in Epidemiology) reporting guidelines and the CHERRIES (Checklist for Reporting Results of Internet E-Surveys) [47,48].

### Ethical Considerations

Ethical approval is granted by the New York University Shanghai institutional review board (2022-008-NYUSH). Participants provided digital informed consent before participating and, upon completing the survey, received a compensation of 6 Chinese Yuan (approximately US $1).

## Results

### Respondent Demographics

A total of 3763 individuals responded to the survey. The analytical sample of this study is 3230 respondents, removing 118 replicants, 156 minors under the age of 18 years, 190 individuals not living in Shanghai at the time of responding to the survey, and 69 individuals who failed to respond correctly to survey validity questions (eg, questions that had 1 correct answer: “Please indicate 2 as the answer to this question”).

Table 1 presents the characteristics and bivariable associations between study covariates and mobile mental health service use intention. The average age of the respondents was 34.4 (SD 10.92) years. The majority of the respondents were male (1657, weighted percentage=55.4%), aged between 25 to 34 years (n=1346, 36.8%), and with a Shanghai hukou (n=1440, 50.1%). Most of the participants had at least good health (n=2964, 92.1%), and only a small portion had a past psychiatric illness diagnosis (n=179, 4.6%). About one-third (n=1030, 29.7%) of the respondents had anxiety or depression: 127 (3.6%) participants had anxiety only, 335 (9.6%) had depression only, and 568 (16.5%) had comorbid anxiety and depression.

**Table 1:** Participant characteristics of Shanghai residents during the 2022 lockdown and bivariable associations with intention to use mobile mental health services (N=3230). Data shown are raw numbers and weighted percentages.

			Mobile mental health service use intention															
			No				Yes					Total						
			Counts, n		%		Counts, n		%		Counts, n			%		*P* value		
Total			2502		80.7		728		19.3		3230			100				
**Gender**																		<.001
	Female	1143		74		420		26		1563			44.4					
	Male	1350		86		307		14		1657			55.4					
	Other	9		93.3		1		6.7		10			0.3					
**Age (years)**																		<.001
	18-24	422		74.8		144		25.3		566			13.1					
	25-34	997		76.1		349		23.9		1346			36.8					
	35-44	607		80.9		153		19.2		760			20.6					
	45-54	318		81.6		65		18.4		383			10.6					
	≥55	158		93.2		17		6.8		175			18.8					
**Ethnicity**																		.47
	Han	2422		80.6		704		19.4		3126			96.9					
	Other	80		83.7		24		16.3		104			3.1					
**Education**																		.001
	Secondary or lower	384		85.6		87		14.4		471			14.7					
	High school	573		84.8		141		15.2		714			22.5					
	College or higher	1545		78.1		500		21.9		2045			62.7					
**Marital status**																		.006
	Single	820		76.8		259		23.3		1079			27.3					
	Married or cohabitating	1579		81.9		453		18.1		2032			68.9					
	Divorced or widowed or other	103		88.1		16		11.9		119			3.8					
**Monthly income**																		.01
	Less than 4000	277		80.9		74		19.1		351			8.7					
	4000-8000	718		84.5		186		15.5		904			31.4					
	More than 8000	1507		78.7		468		21.3		1975			59.9					
**Employment**																		<.001
	Employed	1818		77.4		542		22.6		2360			64.5					
	Unemployed	295		83.9		67		16.1		362			10.3					
	Other	389		87.9		119		12.1		508			25.2					
**Hukou status**																		.45
	With Hukou	1086		81.5		354		18.5		1440			50.1					
	Migrant without Hukou	1416		80		374		20		1790			49.9					
**Loss in income**																		<.001
	None	604		88.9		125		11.1		729			30.8					
	Less than 25%	511		71		217		29		728			20.3					
	Between 25%-50%	496		76.9		169		23.1		665			18.3					
	Between 51%-75%	279		75.7		85		24.3		364			10.6					
	More than 75%	612		84.3		132		15.7		744			20.1					
**Food security**																		<.001
	Food secure	446		91.6		71		8.4		517			17.2					
	Mild food insecure	394		88.1		82		11.9		476			16.4					
	Moderate food insecure	1279		75.8		452		24.21		1731			51.8					
	Severe food insecure	376		76.9		122		23.1		498			14.6					
**Potentially trauma events (PTE)**																		<.001
	No or mild PTE	1791		85.6		373		14.4		2164			67.4					
	Loss of loved one in the past 12 months only	284		75.1		117		24.9		401			12.5					
	PTE in lockdown	427		67.9		238		32.1		665			20.1					
**Emotional support**																		<.001
	No	651		88.6		111		11.5		762			25.5					
	Yes	1851		78.1		617		22		2468			74.5					
**Financial support**																		<.001
	No	1567		84.8		370		15.2		1937			64.5					
	Yes	935		73.3		358		26.7		1293			35.5					
**Food support**																		.85
	No	93		81.7		29		18.3		122			3.4					
	Yes	2409		80.7		699		19.3		3108			96.6					
**Self-rated health**																		<.001
	Fair or poor	180		67.4		86		32.6		266			8					
	Excellent or very good or good	2322		81.9		642		18.1		2964			92.1					
**Past psychiatric illnesses**																		<.001
	No	2388		81.5		663		18.5		3051			95.4					
	Yes	114		64.5		65		35.5		179			4.6					
**Mental health**																		<.001
	No or mild depression or anxiety	1832		86.5		368		13.5		2200			70.3					
	Anxiety only	66		52.1		61		47.9		127			3.6					
	Depression only	254		80.2		81		19.8		335			9.6					
	Anxiety and depression	350		62.6		218		37.4		568			16.5					
**Digital mental health service use**																		<.001
	No	2268		83.7		522		16.3		2790			89.1					
	Yes	48		10.5		392		89.5		440			10.9					

Related to stressors experienced during the lockdown, over half of the respondents (n=2229, 66.4%) experienced at least moderate food insecurity. Most participants experienced lockdown-related loss in income (n=2501, 69.2%), with over 20% (n=744, 20%) experiencing more than 75% loss of income. About 12.4% (401/3230) of the respondents reported having lost loved ones in the past 12 months but did not have potentially traumatic experiences during the lockdown, and one-fifth of the respondents (665/3230, 20.1%) reported that they had potentially traumatic experiences during the lockdown.

For external support during the lockdown, three-fourth of the respondents (2468/74.5) received emotional support, one-third of the respondents (1293/3230, 35.5%) received financial support, and most of the respondents (3108/3230, 96.6%) received food support.

### Intention for the Use of Mental Health Services and Digital Mental Health Services

**Figure 1:**
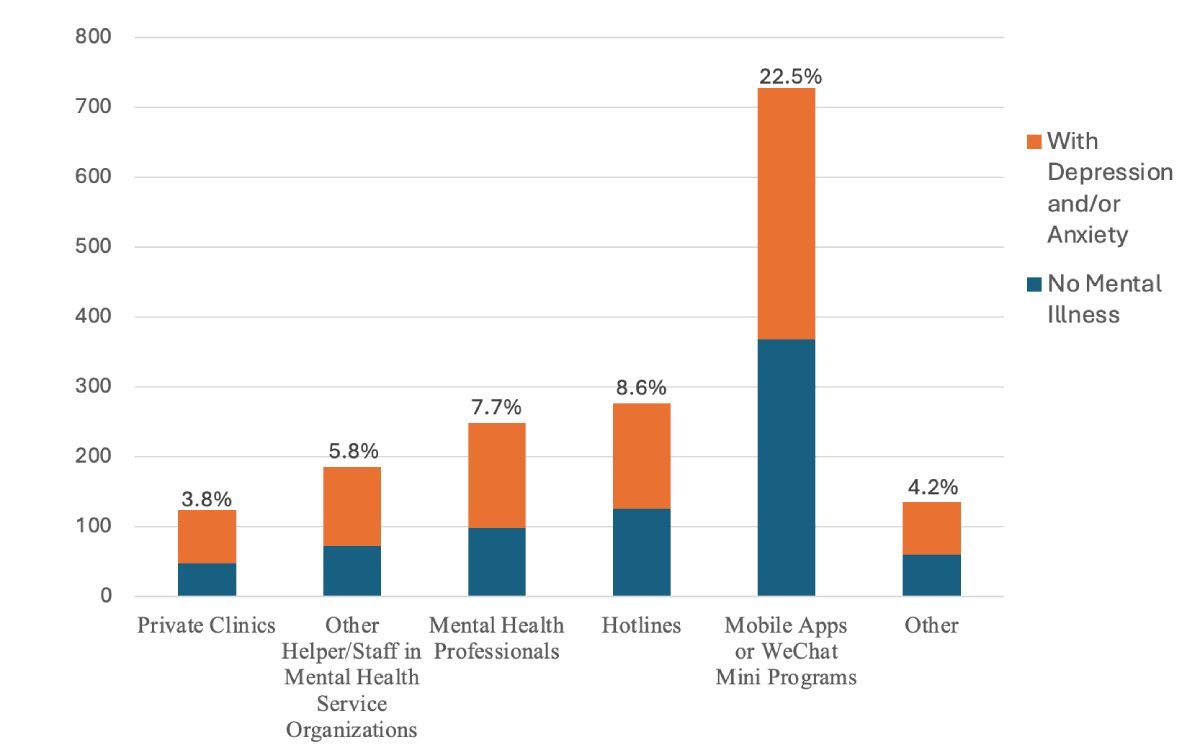
Mental health service use intention by service or provider types (N=3230, with any intent n=914). Percentage reflects the percentage among all qualified respondents.

Figure 1 reports the mental health service use intention by different service types or providers among the respondents. One-fourth of the respondents (n=914, 24.4%) reported that they had considered seeking mental health services during the lockdown. Mobile mental health (mobile apps or WeChat Mini Programs) was the most considered (n=728, 19.3%), and private clinics received the least consideration (n=124, 3.5%). About half of those who indicated service use intention did not screen as having depression and/or anxiety.

Among those who met screening eligibility for depression or anxiety, only 41.2% (n=454) indicated an intention to use any mental health services, and 33% (n=360) indicated an intention to use digital mental health services.

A small portion of the respondents (n=440, 10.9%) reported that they had used digital mental health services. About half (n=216, 47.4%) of these respondents reporting using digital mental health services during the lockdown screened positive for depression or anxiety.

### Factors Associated With the Intention to Use Mobile Mental Health Services

Table 2 presents the logistic regression analysis results, adjusting for demographic covariates, to identify predictors of mobile mental health service use intention during the lockdown. Being male (adjusted odds ratio [aOR] 0.60, 95% CI 0.46-0.77), unemployed (aOR 0.58, 95% CI 0.39-0.85), or a migrant without hukou (aOR 0.69, 95% CI 0.52-0.91) were associated with decreased odds of intention to use services. In terms of covid-related experiences, experiencing moderate food insecurity (aOR 2.35, 95% CI 1.64-3.38), severe food insecurity (aOR 2.01, 95% CI 1.26-3.21), loss of income less than 25% (aOR 1.88, 95% CI 1.29-2.73), potentially traumatic experiences during lockdown (aOR 2.16, 95% CI 1.61-2.89), emotional support (aOR 1.87, 95% CI 1.34-2.61), financial support (aOR 1.42, 95% CI 1.11-1.82), were associated with increased odds of mobile service use intention. In the physical and mental health domains, having moderate to severe anxiety (aOR 4.58, 95% CI 2.46-8.51) and moderate to severe anxiety and depression (aOR 2.68, 95% CI 1.99-3.60) were associated with increased odds of service use intention.

**Table 2:** Associations between lockdown stressors, support, physical, and mental health on mobile mental health service use intent (N=3230).

		Mobile mental health service use intention	
		Odds ratio	95% CI
**Gender**			
	Female	1.00	—^a^
	Male^b^	0.60	0.46-0.77
	Other	0.17	0.02-1.27
**Age categories (years)**			
	18-24	1.00	—
	25-34	0.97	0.63-1.50
	35-44	0.78	0.47-1.29
	45-54	0.87	0.48-1.58
	≥55	0.45	0.17-1.18
**Ethnicity**			
	Han	1.00	—
	Other	0.74	0.39-1.42
**Education**			
	Secondary or lower	1.00	—
	High school	0.96	0.65-1.43
	College or higher	0.96	0.65-1.42
**Marital status**			
	Single	1.00	—
	Married or cohabitating	0.80	0.58-1.11
	Other	0.77	0.35-1.69
**Monthly income**			
	Less than 4000	1.00	—
	4000-8000	0.89	0.60-1.30
	More than 8000	0.99	0.68-1.44
**Employment categories**			
	Employed	1.00	—
	Unemployed^b^	0.57	0.39-0.85
	Other	0.74	0.44-1.24
**Hukou status**			
	With Hukou	1.00	—
	Without Hukou^b^	0.69	0.52-0.92
**Household Food Insecurity Access Scale categories**			
	Food secure	1.00	—
	Mild FI^c^	1.48	0.94-2.33
	Moderate FI^b^	2.35	1.63-3.37
	Severe FI^b^	2.02	1.27-3.22
**Loss in income**			
	None	1.00	—
	Less than 25%^b^	1.88	1.29-2.74
	Between 25%-50%	1.35	0.92-1.97
	Between 51%-75%	1.62	0.91-2.88
	More than 75%	1.01	0.65-1.57
**Experienced PTE^d^**			
	No or mild PTE	1.00	—
	Loss past 12 months^b^	1.91	1.40-2.62
	PTE during lockdown^b^	2.15	1.60-2.88
**Any emotional support**			
	No	1.00	—
	Yes^b^	1.87	1.35-2.60
**Any financial support**			
	No	1.00	—
	Yes^b^	1.43	1.12-1.83
**Any food support**			
	No	1.00	—
	Yes	0.89	0.48-1.64
**Self-rated health**			
	Fair or poor	1.00	—
	Excellent or very good or good	0.72	0.49-1.05
**Past psychiatric illnesses**			
	No	1.00	—
	Yes	1.22	0.82-1.83
**Mental health**			
	No depression or anxiety	1.00	—
	Anxiety only^b^	4.57	2.46-8.46
	Depression only	1.28	0.86-1.90
	Anxiety and depression^b^	2.66	1.98-3.56

^a^Not available.

^b^Estimates are *P*<.05.

^c^FI: food insecurity.

^d^PTE: potentially traumatic events.

## Discussion

### Principal Findings

The COVID-19 pandemic and associated preventative measures, such as city-wide lockdowns, have a significant impact on the mental health of the public [13]. Our study assessed the public’s intention to use mental health services and examined the factors associated with mobile mental health service use intention during the 2022 lockdown among Shanghai residents. Overall, the estimated mental health burden was high, with almost 30% of the respondents meeting screening thresholds for depression or anxiety. Approximately 25% of the respondents reported considering mental health services during the lockdown.

Our results found that mobile mental health programming was the most popular mental health services considered, with about 20% of the respondents considering using these services during the lockdown compared with only 8.6% of the respondents considering hotlines. This is consistent with previous research revealing that people prefer social media and mobile apps over other digital formats for mental health support [49]. Mobile mental health services have several advantages that may be appealing to the general public, including reduced treatment burden (the amount of effort individuals need to spend to receive treatment [50]), protection of privacy, and the capacity to provide services with less stigma [51,52]. However, not all individuals who had service use intention used services. Our preliminary assessment found that only 10.9% of the respondents reported having used digital mental health services (including online classes) during the lockdown, much lower than what has been reported in other countries, suggesting continued underuse of mental health services in China and the development potential of mobile mental health services to fill this critical care gap.

Respondents who considered using mobile mental health services were diverse and were across age groups, income levels, and regions in Shanghai. Previous research has explored the association between age and online information seeking or e-health service use intention. Liu and Shi [22] found that age was not a significant predictor of intention to use virtual health visits among Chinese who were active online information seekers. Similarly, Li et al [53] found that the willingness to use e-hospitals among hospital patients did not differ across ages. Consistent with these findings, we found that age was not a significant predictor of mobile mental health service use intention. This may be attributed to our sample characteristics as Shanghai is one of the largest and most developed cities in China with a high degree of technological integration [21], and online survey respondents also tend to have a higher level of digital literacy. Another important finding is that approximately half of the respondents who indicated considering mobile mental health services had common mental disorders (meeting the screening eligibility for depression or anxiety), while the other half did not meet the screening eligibility for these disorders. This reflects the demands to provide digital mental health services across the care cascade, potentially from prevention to treatment, to meet the needs of diverse target consumers.

This study yielded interesting findings regarding the association between mental health disorders and the intention to use mobile mental health services. Individuals with moderate or severe anxiety or comorbid anxiety and depression demonstrated a higher intention to use these services. However, individuals with depression only did not exhibit a significantly higher intention compared to those without common mental disorders. We also did not observe a statistically significant association between past psychiatric diagnosis and mobile mental health service use intention, which contradicts previous research on general mental health service use intention [27]. One potential explanation relates to psychiatric labeling, a process in which an individual is attached to a diagnosis or label based on their mental health diagnosis, symptoms, or behavior [54]. The ability of individuals to recognize a mental health problem can influence their intentions to seek health care services [55]. Studies have found that people may underestimate their anxiety severity but overestimate their depression severity [56], and those with depressive symptoms may have help-negation [57,58]. Individuals with past psychiatric diagnoses received confirmed labeling from a trained mental health professional, and their preferences for the type of help they seek may also be different compared to those who only self-recognized their symptoms [27]. All these considerations could influence individuals’ intention to seek mobile mental health services. Research has examined various factors, such as self-stigma, perceived service helpfulness, and past help-seeking behaviors, associated with help-seeking preferences [28], but past research has either treated individuals with depression or anxiety as a homogeneous group or examined depression and anxiety separately. Individuals may seek different types of mental health care based on different symptoms and different psychiatric labeling processes. In LMICs, fewer individuals would have received mental health diagnoses from a trained professional compared, which is particularly important to consider when developing digital mental health services and providing screening and referral for digital mental health service consumers.

### Implications

Our study has important implications for increasing the development, acceptance, and use of digital mental health services in China, especially during large-scale infectious disease preventative measures. In response to the COVID-19 pandemic, China’s national guidelines and policies emphasized providing mental health crisis hotlines to the general public, but we found a very low level of intention to use mental health hotlines. Among our study respondents, mobile apps and WeChat Mini Programs were the most considered mental health services during the Shanghai lockdown. This emphasized the need to make the public more aware of remote mental health services, destigmatize mental health hotlines, and provide regulated and high-quality mobile mental health services in China.

Our results highlighted vulnerable populations that may or may not consider mobile mental health services during a lockdown. Being male, unemployed, and a migrant without hukou residency statuses were particularly at risk as they were less likely to consider mobile mental health services despite their greater mental health burden. Those who received social or financial support were more likely to consider mobile mental health services, suggesting a window of opportunity to boost help-seeking intention through providing various support during lockdowns.

This study also presents the results that show that those who would consider mobile mental health services during lockdown were diverse in terms of demographic characteristics. Previous research conducted in China tested digital mental health interventions in younger adults such as college students [20,59]. Our research findings suggest an opportunity to develop and evaluate digital interventions among diverse age groups, including older adults, who are often left out of digital innovations. Our findings also indicate that individuals with different characteristics, experiences, or symptoms may have different preferences and care usage intentions. Therefore, the coproduction of digital mental health services is crucial for developing person-centered care [59,60].

### Limitations and Strengths

This study has several limitations. First, the study used an online survey platform for survey administration, and most of the respondents were young and educated. This led to selection bias and limited the generalizability of the study findings. To mitigate this, we conducted stratified purposive sampling and generated survey weights to reduce survey response bias. Second, the study used cross-sectional self-reported measures, and the results of the study should not be used to imply causal relationships between the assessed constructs. Third, for survey fatigue considerations, the study only assessed mental health service use intention and did not assess awareness, attitudes, and mental health–related stigma to further explain participants’ preferences. However, previous literature showed that intention is one of the strongest predictors of actual technology use, and attitudes towards technologies or mental health services are not necessarily associated with intention [61-63]. The intention to use mental health services itself is an important construct and could inform the development of targeted individual and group-level interventions that directly target increasing intention [64].

Despite these limitations, the study has several strengths that contribute to the existing research literature. First, to the best of our knowledge, this is the first study to describe patterns and predictors of mental health service use intention and to understand the mental health gap from a geographically representative sample during a city-wide lockdown. This can specifically aid in developing services that are fit for diverse population needs. Second, the study assessed a variety of experiences during the lockdown, such as changes in income, food insecurity, potentially traumatic experiences, and external support. These lockdown-related experiences will help us understand the impact of COVID-19 control policies on the participants.

### Conclusions

During the 2022 Shanghai lockdown, the prevalence of depression and anxiety was high among Shanghai residents. Mobile mental health was the most frequently considered type of mental health service and was considered by a diverse population across age groups, levels of education, and income levels. Stressors during the lockdown, social support, and financial support were associated with increased service use intention. Anxiety or comorbid anxiety and depression were stronger predictors than having depression alone. Vulnerable populations, such as those unemployed and migrants without hukou, may have lower mental health service use intention and should be a focus of tailored intervention and outreach while developing digital mental health services for the pandemic response and beyond.

## Abbreviations

aOR: adjusted odds ratio
CHERRIES: Checklist for Reporting Results of Internet E-Surveys
E-HITS: Extended-Hurt/Insult/Threaten/Scream
GAD-7: 7-item Generalized Anxiety Disorder Scale
HFIAS: Household Food Insecurity Access Scale
LMIC: low- and middle-income country
PHQ-9: 9-item Patient Health Questionnaire
PTE: potentially traumatic experiences
STROBE: Strengthening the Reporting of Observational Studies in Epidemiology
